# Phase Transitions and Formation of a Monolayer-Type Structure in Thin Oligothiophene Films: Exploration with a Combined In Situ X-ray Diffraction and Electrical Measurements

**DOI:** 10.1186/s11671-019-3009-8

**Published:** 2019-05-30

**Authors:** Eduard Mikayelyan, Linda Grodd, Viachaslau Ksianzou, Daniel Wesner, Alexander I. Rodygin, Holger Schönherr, Yuriy N. Luponosov, Sergei A. Ponomarenko, Dimitri A. Ivanov, Ullrich Pietsch, Souren Grigorian

**Affiliations:** 1 Department of Physics, University of Siegen, Walter-Flex-Strasse 3, 57072 Siegen, Germany; 2 Department of Engineering and Natural Sciences, Technical University of Applied Sciences Wildau, Hochschulring 1, 15745 Wildau, Germany; 3 Physical Chemistry I, Department of Chemistry and Biology & Research Center of Micro and Nanochemistry and Engineering (Cμ), University of Siegen, Adolf-Reichwein-Strasse 2, 57076 Siegen, Germany; 4 Faculty of Fundamental Physical and Chemical Engineering, Lomonosov Moscow State University, GSP-1, Leninskie gory1, Moscow, Russian Federation 119991; 5 Moscow Institute of Physics and Technology (State University), Institutskiy per. 9, Dolgoprudny, Russian Federation 141700; 6 Enikolopov Institute of Synthetic Polymeric Materials of Russian Academy of Sciences, Profsoyuznaya st. 70, Moscow, Russian Federation 117393; 7 Chemistry Department, Lomonosov Moscow State University, Leninskie Gory 1-3, Moscow, Russian Federation 119991; 8 Institut de Sciences des Matériaux de Mulhouse (CNRS UMR 7361), 15 rue Jean Starcky, B.P 2488, 68057 Mulhouse, France; 9 Institute of Problems of Chemical Physics, Russian Academy of Sciences, Chernogolovka, Moscow region, Russian Federation 142432; 10 Aix Marseille University, University of Toulon, CNRS, IM2NP, Campus de St-Jérôme, 13397 Marseille, France

**Keywords:** Thin films, Interfacial monolayers, Oligomers, Quarterthiophenes, In situ GIXD, Phase transitions, Mesophase

## Abstract

A combination of in situ electrical and grazing-incidence X-ray diffraction (GIXD) is a powerful tool for studies of correlations between the microstructure and charge transport in thin organic films. The information provided by such experimental approach can help optimizing the performance of the films as active layers of organic electronic devices. In this work, such combination of techniques was used to investigate the phase transitions in vacuum-deposited thin films of a common organic semiconductor dihexyl-quarterthiophene (DH4T). A transition from the initial highly crystalline phase to a mesophase was detected upon heating, while only a partial backward transition was observed upon cooling to room temperature. In situ electrical conductivity measurements revealed the impact of both transitions on charge transport. This is partly accounted for by the fact that the initial crystalline phase is characterized by inclination of molecules in the plane perpendicular to the π-π stacking direction, whereas the mesophase is built of molecules tilted in the direction of π-π stacking. Importantly, in addition to the two phases of DH4T characteristic of the bulk, a third interfacial substrate-stabilized monolayer-type phase was observed. The existence of such interfacial structure can have important implications for the charge mobility, being especially favorable for lateral two-dimensional charge transport in the organic field-effect transistors geometry.

## Introduction

Organic semiconductors constitute an important class of materials due to their exceptional combination of mechanical flexibility and low cost allowing production of large-area electronic devices. They are used as functional layers in various organic electronic circuits such as organic field-effect transistors (OFETs), organic light emitting diodes (OLEDs), organic photovoltaics (OPVs), and others [1, 2]. Understanding the relation between the active layer structure and device properties is crucial for the optimization of the performance of the devices based thereupon. One of the common techniques for the structural analysis of organic semiconductor materials is X-ray diffraction. In particular, grazing-incidence X-ray diffraction (GIXD) using intense synchrotron X-ray beams is a powerful tool providing sensitivity to organization of the interfacial regions of the organic films close to the substrate and probing the thicknesses on the order of a few monolayers that are mainly responsible for the charge transport.

For optimization of the performance of such devices as organic field-effect transistors (OFETs), it is important to consider that the structure of thin films can significantly differ from that of bulk single crystals [3]. Commonly, organic semiconductor films cast on substrate form crystallites, which may be randomly oriented with respect to the substrate surface. If the crystallite orientation is random in 3D, Bragg diffraction peaks corresponding to same *d*-value form a ring-like pattern. If the random orientation is restricted to the plane parallel to the substrate, well-defined Bragg spots appear allowing to analyze the film texture. Consequently, in the case of sufficiently intense diffraction peaks, 2D-GIXD is a suitable technique for in situ investigations of thin-film structures during various processes such as solidification and post-annealing [4–6]

Generally, the rod-like oligothiophene molecules deposited on substrates exhibit nearly upright orientation, with the long molecular axis being almost perpendicular to the substrate surface [7]. Accordingly, the π-π stacking direction is largely oriented parallel to the substrate surface, which is advantageous for the OFET geometry. For utilizing in solution-processable electronics, the solubility improvement by aliphatic end group substitution is common [8, 9]. It is known that the increasing number of thiophene units increases the charge carrier mobility at the cost of decreasing the solubility. For this reason, the optimum length of the thiophene core is considered to be quarterthiophene (4T) [10].

Oligothiophenes are the most studied organic semiconductor materials [11]. These rod-like molecules provide a relatively high mobility in thin films caused by preferential π-π stacking [12] and are promising for applications in organic electronics [13–15]. Dihexyl-quaterthiophene (DH4T) is one of the well-known oligothiophenes [16–20]. Based on differential scanning calorimetry (DSC), two endotherms were reported, one at 81 °C and the other at 181 °C, where the first one is conventionally attributed to a transition to the mesophase and the second one to the isotropization [10, 21, 22]. Previously, the monoclinic structure of single D4HT crystals was analyzed by electron diffraction [23]. Furthermore, annealing of DH4T fibers revealed two crystallographic phases corresponding to the initial phase and the mesophase [10]. In the case of thin films [21], the structure of the mesophase was associated with a tilted pseudohexatic smectic structure, whereas in the study of fibers, it was identified as a crystalline phase II [10].

Apart from the rich polymorphism in the bulk, the considered organic molecules are often prone to formation of the so-called surface-induced polymorphs, or surface-mediated polymorphs [24, 25]. In this case, nucleation occurs in the proximity of a surface and results in a structure different from any of the bulk polymorphs. Such surface-induced structures can be very important for the charge transport properties of the functional films.

In this work, we report combined temperature-resolved studies of the phase transitions of vacuum-deposited DH4T films. The observed structural changes before and after phase transitions are correlated with electrical conductivity, and the implications of the interfacial region organization on the charge transport are discussed.

## Methods

### Materials

A sample of 5,5‴-dihexyl-2,2′:5′,2″:5″,2‴-quarter-thiophene (DH4T) was prepared similar to the method described elsewhere [26]. The product was purified by recrystallization from toluene/hexane mixture to give 647 mg (65%) of yellow crystals. Molecular structure and purity of the final product was proved by 1H NMR spectroscopy and elemental analysis. ^1^H NMR (250 MHz, CDCl_3_, TMS/ppm): 0.89 (t, 6H, *J* = 6.7 Hz), 1.23–1.45 (overlapped peaks, 12 H), 1.67 (m, 4H), 2.78 (t, 4H, *J* = 7.3 Hz), 6.67 (d, 2H, *J* = 3.7 Hz), 6.96 (d, 2H, *J* = 3.4 Hz), 6.99 (d, 2H, *J* = 3.7 Hz), 7.01 (d, 2H, *J* = 3.7 Hz). Calc.for C_28_H_34_S_4_: C, 67.42; H, 6.87; S, 25.71. Found: C, 67.31; H, 6.91; S, 25.66%.

### Sample Preparation

As a substrate, doped Si with thermally grown 230 nm SiO_2_ layer was used. Prior to DH4T material evaporation, the substrates were cleaned in Piranha solution to remove all organic contaminations and obtain hydrophilic surface; it was further washed with distilled water and dried in a nitrogen stream afterward. The DH4T semiconductor was thermally evaporated in a vacuum deposition chamber under high vacuum at 10^−6^ mbar with the evaporation rate of 0.2 Å/s fixed by PID controller. The material was deposited on the substrate at room temperature.

### X-ray Characterization

Grazing-incidence X-ray diffraction experiments were performed at P08 beamline of the PETRA III synchrotron of DESY (Hamburg, Germany) and BL9 beamline of the DELTA synchrotron (Dortmund, Germany). At P08 beamline, the employed X-ray micro-beam had the dimensions of 20 × 60 μm^2^ in horizontal and vertical directions, respectively. The photon energy of 20 keV was used to reduce the radiation damage of the organic films. The micro-beam was incident on the 18 × 18 mm^2^ samples at an angle of *α*_i_ = 0.07°. The Perkin Elmer (XRD1621) flat panel was used to record the diffraction patterns. Diffraction images of 2048 × 2048 pixels were acquired with the pixel size of 200 μm in both horizontal and vertical directions. At BL9 beamline of the DELTA synchrotron, the beam with the energy of 15 keV and dimensions of 0.2 × 1 mm^2^ was utilized. The incidence angle *α*_i_ was of 0.1°. The diffraction patterns were recorded by Mar image plate with 3450 × 3450 pixels having the pixel size of 100 μm.

The sample annealing was performed with a Linkam heating stage (HFSX350-GI) adapted for the grazing-incidence geometry. The heating rate used during the heating ramps was equal to 30 °C/min. Prior to X-ray exposure, the sample was equilibrated for 3 min at each measurement temperature.

X-ray reflectivity (XRR) curves were obtained using Cu Kα radiation at the in-house STOE reflectometer. Both diffraction and reflectivity measurements were performed under ambient conditions.

### AFM Characterization

Atomic force microscopy (AFM) height images were obtained in intermittent contact (tapping) mode on an Asylum Research MFP-3D Bio AFM instrument (Asylum Research, Santa Barbara, CA) using AC 160 TS silicon cantilevers with a nominal spring constant of 26 N/m (Olympus, Tokyo, Japan). Images were taken with a resolution of 512 × 512 pixels at a scan rate of 1.0 Hz. All data were acquired at ambient temperature and pressure.

### Electrical Characterization

The electrical conductivity measurements on the vacuum-evaporated films were carried out on a Keithley’s 2612A SourceMeter. This device allows to simultaneously applying two voltage signals and measuring two corresponding current responses. Electrical characterization has been done with the help of the OFET testbeds commercially available from Fraunhofer IPMS, Dresden, Germany. We have employed a custom made setup using special gold-sputtered metal pins with springs to connect to the contact pads of the interdigitated OFETs in the bottom contact geometry with the channel length of 20 μm and channel width of 10 mm.

## Results and Discussion

**Fig. 1:**
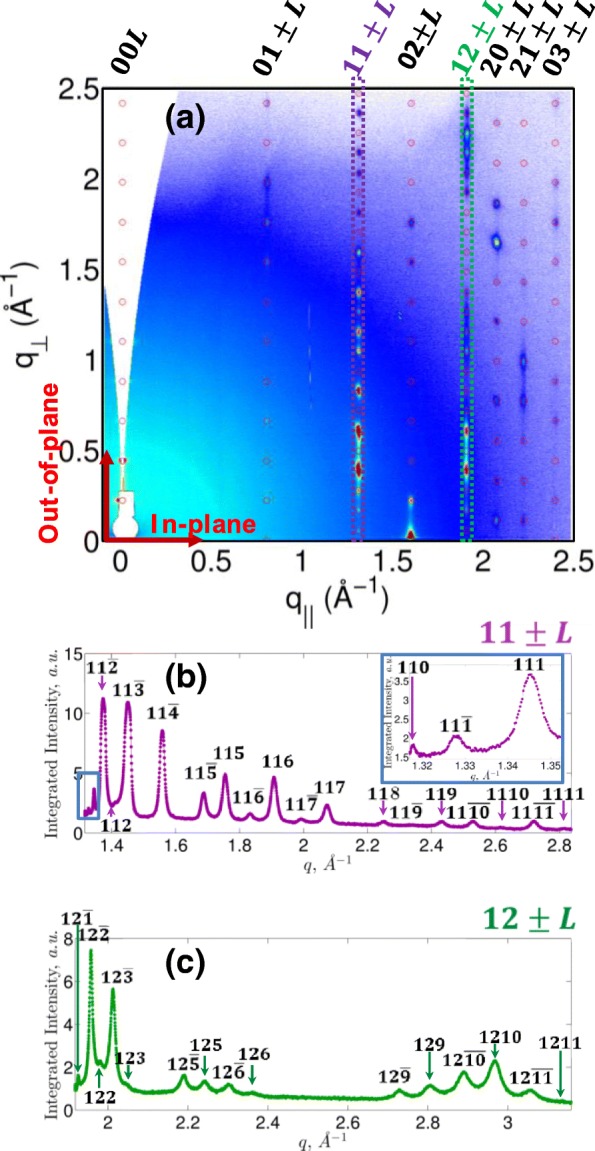
**a** 2D-GIXD patterns of the highly crystalline vacuum-deposited DH4T film with overlaid simulated Bragg reflections (red circles) for a monoclinic unit cell. Intensity line profiles measured along *q*_⊥_of **b** 11 ± *L* and **c** 12 ± *L* reflection families are given in purple and green color, respectively

Thin DH4T films were prepared by vacuum deposition at room temperature on a Si/SiO_2_ substrate. The diffraction patterns of DH4T thin films were measured by in situ GIXD as a function of temperature. The angularly resolved data were converted to reciprocal space where the axes of perpendicular (*q*_⊥_) and parallel (*q*_‖_) components of the momentum transfer vector correspond to scattering along perpendicular (out-of-plane) and parallel (in-plane) directions, respectively. The converted reciprocal space map of DH4T film at 30 °C is given in Fig. 1a.

In total, more than 70 Bragg reflections were observed in the GIXD patterns of DH4T thin films. The proposed indexation of reflections (cf. Fig. 1a–c and text below) demonstrates that the initial thin-film structure is highly crystalline and uniformly oriented with respect to the film surface. The large number of reflections recorded by means of a Perkin Elmer 2D detector at the P08 beamline [27] of the PETRA III synchrotron (DESY, Hamburg) makes it possible to determine the unit cell parameters via structural modeling. The suggested model is described by a monoclinic unit cell.

The fitted Bragg reflection positions in reciprocal space are overlaid on the measured diffraction pattern (Fig. 1a). The out-of-plane direction is parallel to the c* vector, i.e., assigned to the Miller *l* index, whereas the in-plane indices are *h* and *k*. In our analysis, we performed sections of the 2D diffraction patterns along different crystallographic directions. Thus, Fig. 1 b and c show the sections along the so-called truncation rods of highest intensities traced through reflections 11 ± *l* (i.e., 110, 11-1, 111) and 12 ± *l* where index *l* varies from zero through eleven as reflections up to the 11th order in *l* could be observed. The calculated positions of the reflections for the D4HT film at room temperature result in the following monoclinic unit cell: *a* = (6.0 ± 0.1) Å, *b* = (7.8 ± 0.1) Å, *c* = (28.5 ± 0.1) Å, and *β* = (93 ± 1)°. The analyzed film texture corresponds to the (**ab**) plane parallel to the plane of the substrate. The comparison of the unit cell parameters in current study with previously reported structural data from DH4T taken from single crystal [23] and fibers [10] is summarized in Table 1. It can be seen that the unit cell parameters of the film studied in the present work are rather close to those of the bulk phases addressed previously. This fact can result from the high quality of the evaporated film formed at very low deposition rates.

**Table 1:** The unit cell parameters for the DH4T oligothiophene for the room-temperature crystalline phase

Sample	Monoclinic unit cell parameters
Evaporated DH4T	*a* = 6.0 Å, *b* = 7.8 Å, *c* = 28.5 Å, and *β* = 93.0°
Single crystal of DH4T [23]	*a* = 6.049 Å, *b* = 7.814 Å, *c* = 28.532 Å, and *β* = 92.39°
Fibers of DH4T [10]	*a* = 6.09 Å, *b* = 7.81 Å, *c* = 28.49 Å, and *β* = 91.9°

**Fig. 2.**
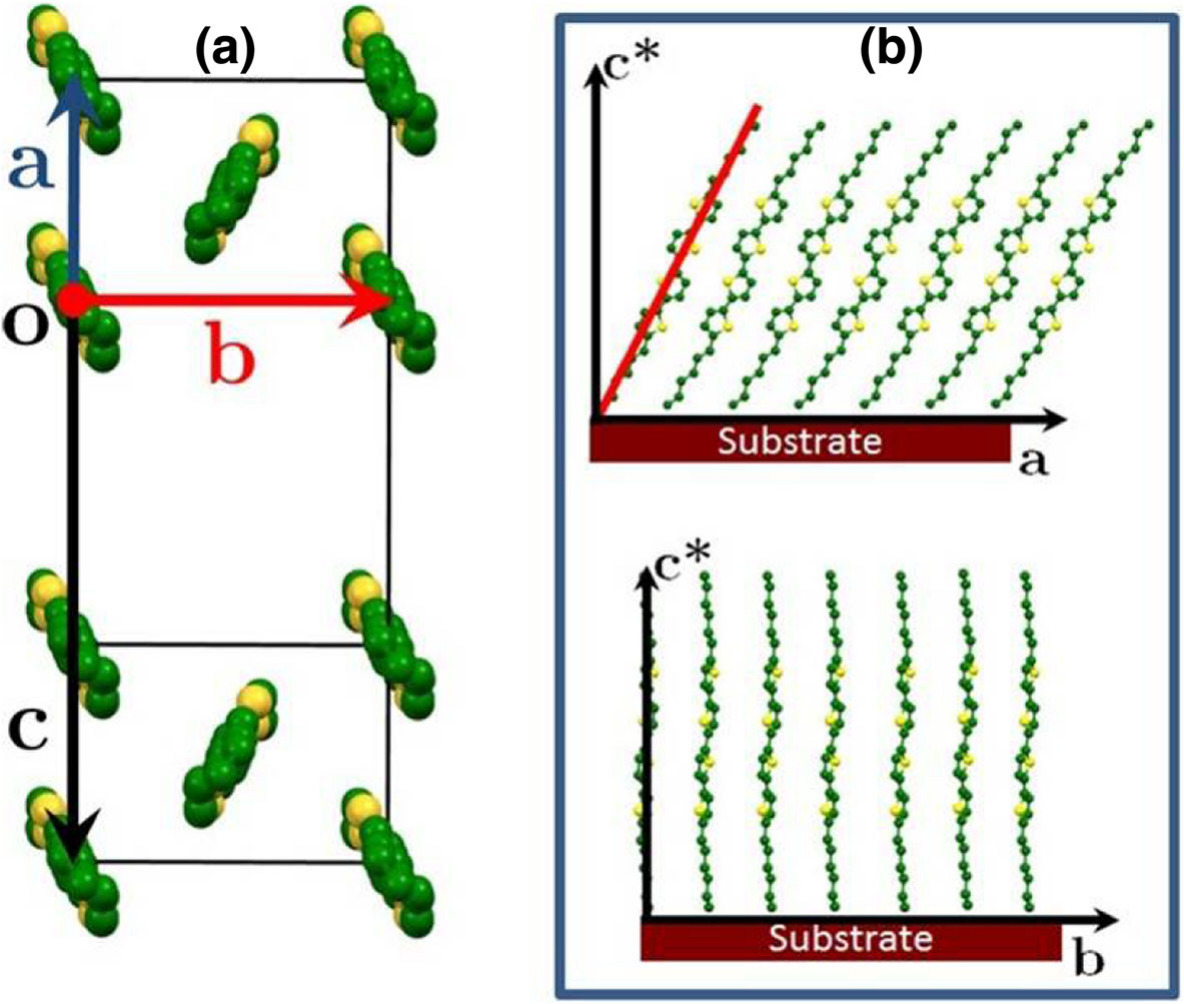
**a** Unit cell structure of the DH4T compound in the low-temperature crystalline phase viewed along the molecular direction and **b** orientation of the molecules with respect to the substrate surface

The molecular orientation with respect to the unit cell is shown in Fig. 2a. When looking along the longest dimension of the molecules, the characteristic herring-bone arrangement of the thiophene blocks can be observed. Moreover, since the 020 reflection is associated to the stacking of the π-π orbitals, the observation of the 020 reflection in the in-plane direction presumes that the molecule in the unit cell does not exhibit a tilt in the direction of π-π stacking. On the other hand, the tilt in the direction perpendicular to the π-π stacking is noticeable, as shown in Fig. 2b.

The intensities calculated for the 11 ± *l* and 12 ± *l* reflection families fit well the evaporated thin-film structure. The molecular inclination with respect to the film normal can be estimated as follows. Indeed, the d-spacing corresponding to the 001 reflection for the monoclinic unit cell is *d*_001_ = *c* sin*β*. On the other hand, the tilt angle *Θ*_*t*_ of the molecule with respect to the substrate normal is *Θ*_*t*_ = cos^−1^(*d*_001_*/l*), where *l* is the calculated molecular length along the long axis of the molecule (the molecular length of DH4T is calculated to be 32.5 Å). Hence, the inclination angle of the DH4T molecules with respect to the film normal is 29°, which is rather close to that of the single crystal [23]. In comparison, the tilt angle of the D4HT molecules in fibers was reported to be 22° [10].

**Fig. 3:**
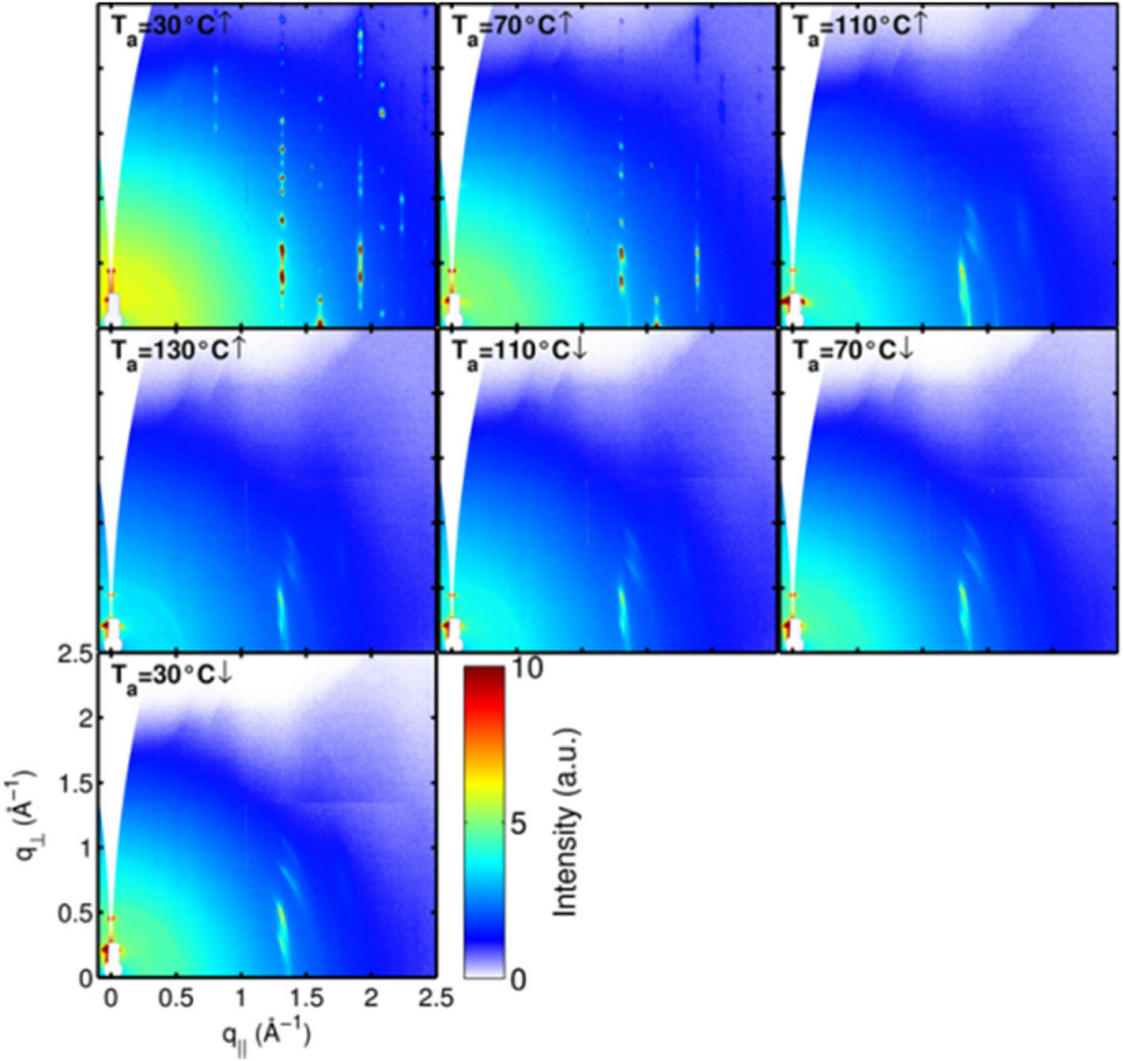
2D-GIXD patterns of the DH4T film acquired at different temperatures

Upon completion of the structure analysis at room temperature, we annealed the samples by raising the temperature up to 130 °C in order to monitor the phase transitions. The 2D-GIXD patterns at different annealing temperatures are shown in Fig. 3. The highly crystalline film is maintained up to 70 °C. Compared to the structure at room temperature, the *c*-parameter remains unchanged, whereas both *a*- and *b*-parameters are increased on 0.1 and 0.2 Å, correspondingly.

The modification of the DH4T structure with temperature can be analyzed in more detail if one takes into account the fact that the alkyl chains and thiophene blocks contribute differently to the different diffraction peaks. Thus, the intensity of the 02L peak series with the exception of 020 and 021 peaks is largely due to the diffraction from the hexyl tails while most of the intensity of the 11L and 12L peaks come from the thiophene blocks. When comparing the DH4T X-ray patterns measured at 30 °C and 70 °C (see Fig. 3), one can notice that the 02L peaks lose intensity faster than the 11L and 12L peaks. This can be explained by the growing concentration of structural defects in the aliphatic regions of the crystal compared to the more ordered thiophene regions, similarly to what has been described in the work of Anokhin et al. [10]. Therefore, one can view this system as being partly disordered under the effect of heating. It is noteworthy that the interactions between the alkyl chains are weak because they are of the London type [28], whereas the thiophenes having sufficient conjugation lengths interact also via stronger π-π interactions [29]. The strength of the chain interaction in unsubstituted oligothiophenes is manifested for example by the melting point, which rapidly grows with molecular weight.

**Fig. 4:**
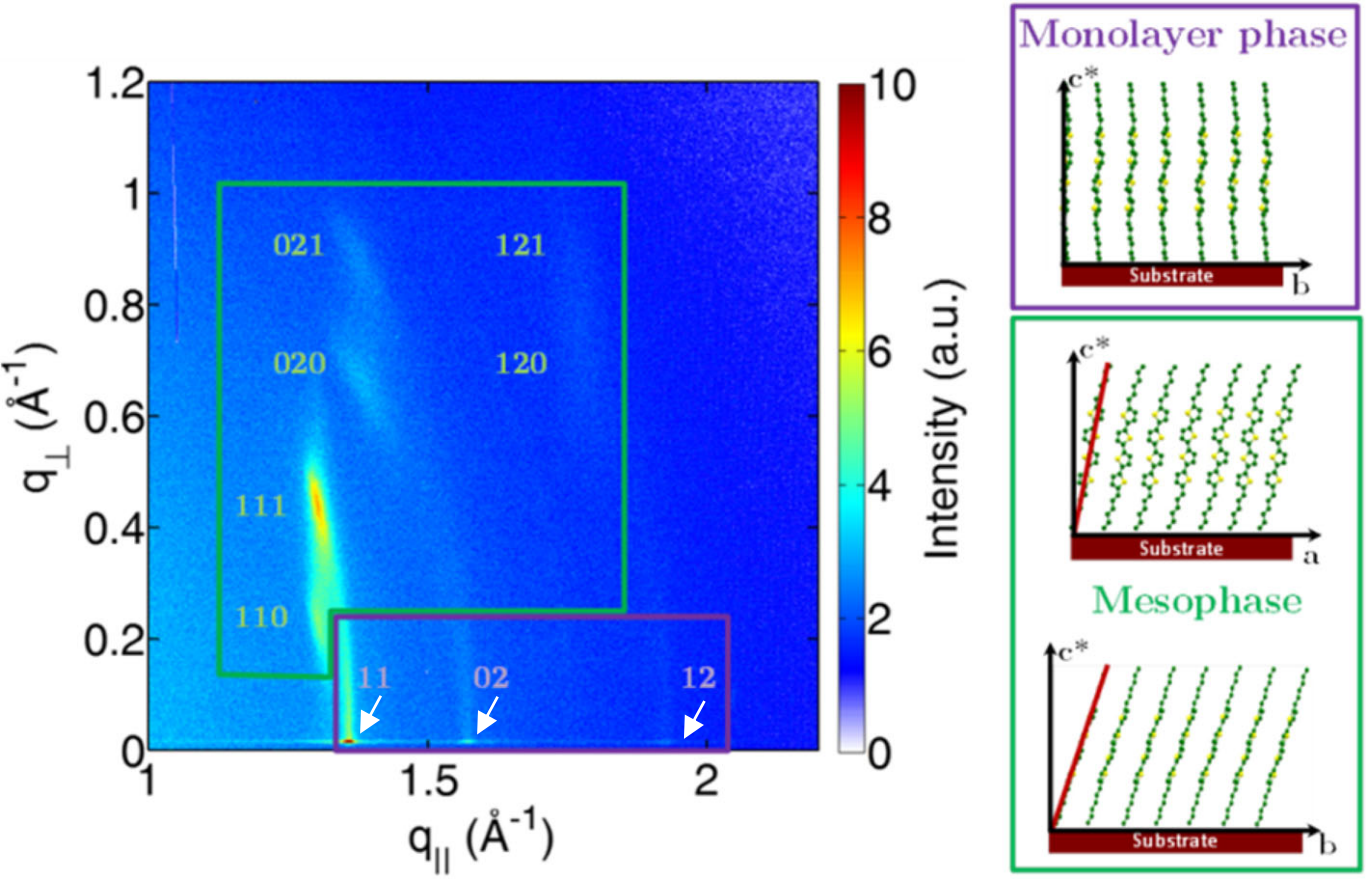
2D-GIXD pattern of a DH4T film measured at 110 °C

By further increasing the temperature up to 110 °C, one can observe a structural transition from the low-temperature highly crystalline phase to a new phase (Fig. 3) that can be identified as a mesophase. Such mesophase was also introduced based on the optical microscopy observations [21]. A single crystalline form was found for the even-numbered α-oligothiophene films evaporated at the low substrate temperatures, whereas the odd-numbered α-oligothiophenes form two different crystalline polymorphs [30]. A monolayer phase on top of the substrate was observed for the vacuum evaporated of α,α′-dihexyl-quinquethiophene (DH5T) and exhibited higher crystallinity at the lover substrate deposition temperatures [31]. Moreover, from the high-temperature 2D-GIXD pattern, it is possible to extract a very interesting piece of structural information. Indeed, at this temperature, in addition to the bulk mesophase having peaks at *q*_⊥_ ≠ 0 Å^−1^ (marked with the green box in Fig. 4), one can also identify a very particular monolayer-type phase with three in-plane peaks having their maxima at the Yoneda horizon (marked by the purple box in Fig. 4).

The pattern infers the presence of two polymorphs: the first one is associated to a monolayer-type phase with fully upright molecules (purple box) and the thickness of 30 Å, whereas a second polymorph identified as a mesophase (green box). The molecular orientations in the monolayer-type phase and mesophase are illustrated in Fig. 4.

For the monolayer-type phase, the parameters of the 2D Bravais lattice can be computed based on the three in-plane peaks indexed as 11, 02, and 12 and lead to the following: *a* = (5.7 ± 0.1) Å, *b* = (8.0 ± 0.1) Å, and *γ* = (90 ± 1)°. These parameters are in accordance with the structures of monolayers formed by rod-like molecules such as substituted quinquethiophene [32, 33], pentacene [34], and diphenylbithiophene-based rod-like molecule [35]. The structure is ascribed to a phase in contact with the substrate surface, which gives rise to the appearance of truncation rods in the out-of-plane direction. The 02 reflection of the phase in question is completely in-plane, showing that there is no molecular tilt along the π-π stacking direction (Fig. 4). Interestingly, this phase is also detected at 70 °C ↑ (Fig. 3) where weak intensity of the 11 rod is observed. Observation of such a monolayer-type phase can have important implications for the charge mobility measurements because the electrical parameters measured in the OFET geometry are largely determined by the properties of this interfacial phase.

The Bravais lattice parameters for the mesophase were calculated from the in-plane momentum transfer of the 110, 020, and 120 reflections and found to be *a* = (5.7 ± 0.2) Å and *b* = (9.0 ± 0.2) Å at *γ* = (91 ± 2)°. The position of the 020 reflection at *q*_⊥_ ≠ 0 Å^−1^ elucidates the tilt of the molecules in the direction of π-π stacking, which is calculated in our case to be *Θ*_*π-π*_ = (26 ± 2)°. From the 002 reflection (as the strongest 001 reflection was covered by beamstop), it is straightforward to calculate the overall tilt of the molecule in the out-of-plane direction. Since the latter is cumulative in both directions along (*Θ*_*π-π*_) and perpendicular to the π-π stacking direction (*Θ*_⊥(*π-π*)_), the value of *Θ*_⊥(*π-π*)_ can be found as ${\cos}^{-1}\frac{d_{001}}{l\cos {\varTheta}_{\pi -\pi }}$ = 17°. The sketch showing the molecular inclination is given in Fig. 4 (right).

**Fig. 5:**
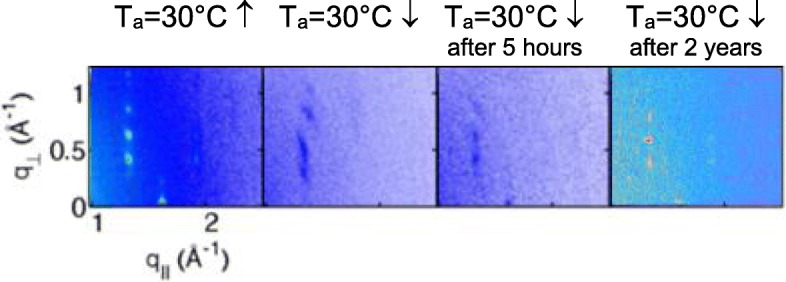
Zoomed 2D-GIXD patterns of the pristine crystalline film: the one measured directly after annealing experiment, as well as the ones kept for 5 h and 2 years at room temperature (from left to right)

When increasing the temperature further, i.e., up to 130 °C, the peak intensities of the monolayer-type structure strongly reduce and only the mesophase structure remains observable. The final structure (30 °C ↓) reveals the following 2D Bravais lattice parameters: *a* = (6.0 ± 0.2) Å, *b* = (9.2 ± 0.2) Å, and *γ* = (95 ± 2)°. Upon fast cooling, the transition from mesophase to the initial crystalline structure does not take place immediately. Thus, it was found that on the time scale of a few hours after cooling down to room temperature, the 2D-GIXD pattern reveals again the structure containing the two polymorphs: the highly ordered crystalline phase and the mesophase. This confirms that on a longer timescale, the conversion of the mesophase to the crystalline phase does take place indeed. However, the backward transition is not complete after 5 h of annealing at room temperature (cf. Fig. 5). However, the measurement conducted after 2 years of annealing at room temperature confirms its full reversibility (cf. right panel of Fig. 5). In this case, the diffraction pattern reveals again the highly ordered crystalline phase typical of the pristine sample.

**Fig. 6.**
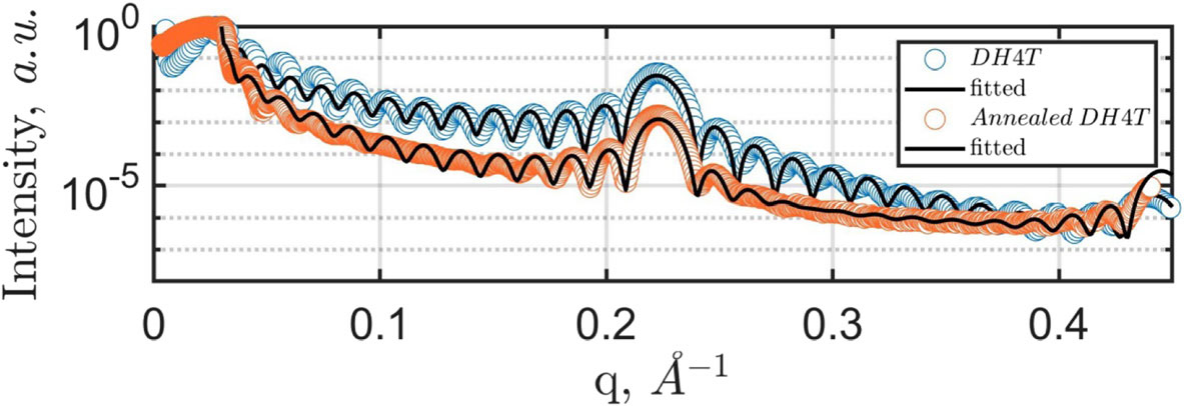
Room-temperature XRR curves of a DH4T film before and after annealing. The dwelling time of the annealed film at room temperature was a week

The multilayered structures were characterized by X-ray reflectivity (XRR). The XRR curves before and after the annealing experiment are shown in Fig. 6. XRR simulations were performed with Motofit package using the Abeles matrix/Parratt recursion and least squares fitting (Genetic algorithm or Levenberg Marquardt). It works in the IGOR Pro environment (TM Wavemetrics) [36]. For simulation, a DH4T monolayer has been subdivided in three sublayers: two identical sheets of hexyl chains with the thickness of 7 Å and a 14-Å-thick layer of 4T-fragments in between. Similar triple sublayer model was introduced in [37] for XRR analysis of benzothiophene films. The sharpness of the air-to-sample and sample-to-substrate interfaces is clearly observable by the Kiessig fringes over the whole q-range of the measurement. The distance between the fringes provides information on the total film thickness while the Bragg peak at *q* = 0.223 Å^−1^ is related to the single-layer thickness. In contrast, the XRR curve obtained for the annealed DH4T film days after annealing experiment shows less pronounced Kiessig fringes revealing an increase of surface and interface roughness of the film from the initial 2–3 to 5–6 Å. The parameters extracted from the XRR analysis are summarized in Table 2.

**Table 2:** Structural parameters extracted from the XRR fits for the film before and after thermal annealing

Sample	Total film thickness	Monolayer thickness	Number of layers
DH4T	(40 ± 1) nm	(28.2 ± 0.1) Å	14
Annealed DH4T	(39 ± 1) nm	(28 ± 0.1) Å	14

**Fig. 7:**
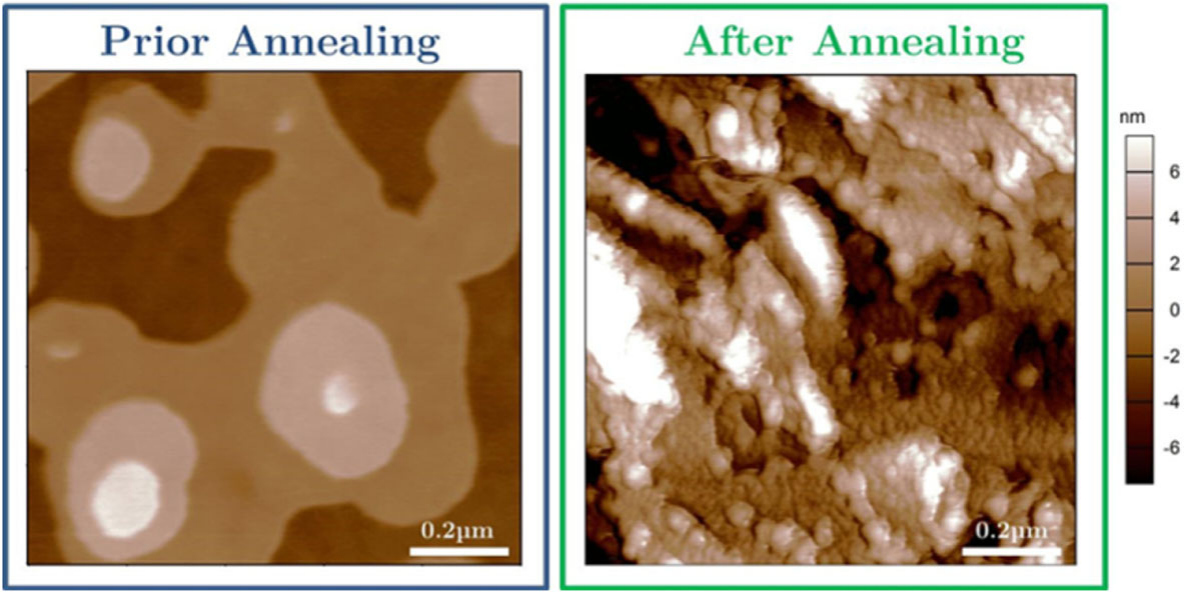
AFM height images of evaporated DH4T acquired at room temperature before and after annealing

The morphology of the film before and after annealing was also examined by atomic force microscopy (AFM). Figure 7 displays height images of the films recorded before and 5 days after the annealing experiment from 1 mm^2^ surface area. Prior to annealing, a highly ordered structure was observed with a very distinct layering within the islands where the height distribution revealed a monolayer thickness which is in qualitative agreement with the XRR data and matches the calculated molecular length of 32.5 Å. In contrast, a very rough morphology of the film was obtained after the annealing, which also confirms the findings of the XRR technique.

**Fig. 8:**
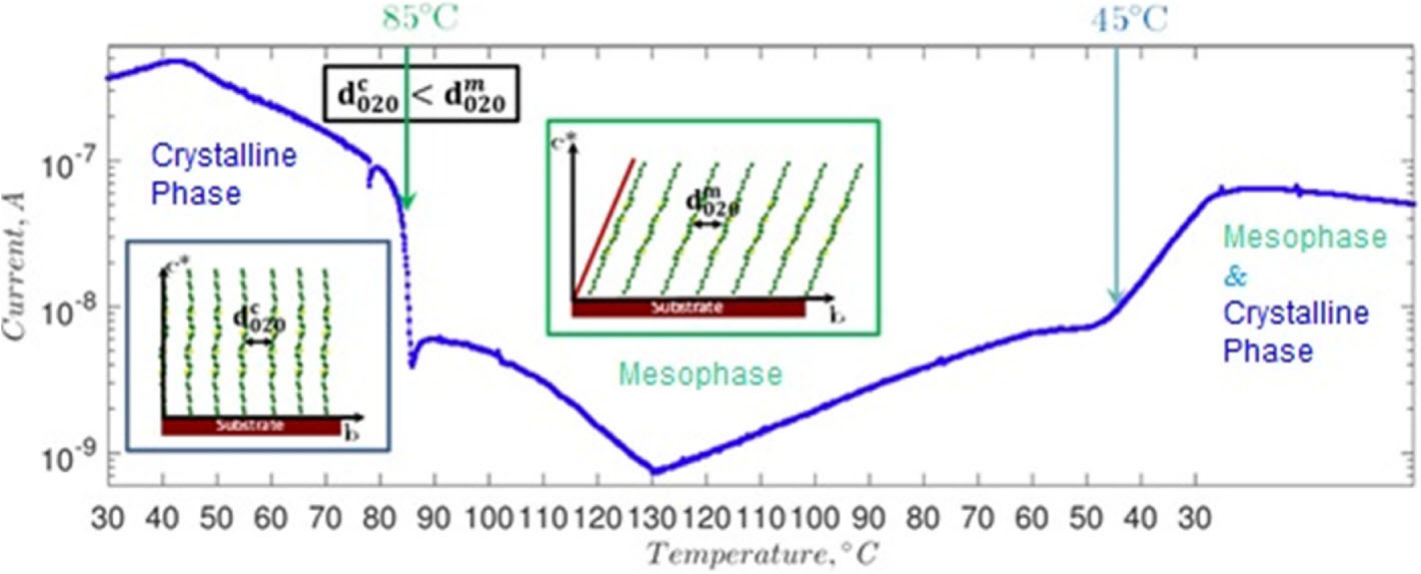
Real time in situ conductivity measurements on a DH4T film during thermal annealing

In our previous studies [10, 31], we reported the mobility values for oligothiophenes with the linear alkyl groups ranging from 0.0004 to 0.08 cm^2^ V^−1^ s^−1^. In the current study, we mainly focus on real-time correlation of the structural and electrical properties. In order to correlate the structure with the electrical performance in OFET geometry, the conductivity measurements were performed during the annealing experiment. The results of real-time in situ analysis are displayed in Fig. 8. The phase transition from the initial crystalline phase to the mesophase was observed at 85 °C, which is visible as a pronounced drop of the current. This can be accounted for by the increase of the π-π stacking distance occurring across the phase transition. Further decrease of conductivity was recorded with an increase of the annealing temperature up to the maximum temperature of 130 °C, for which the lowest conductivity was observed, assigned to the lowest crystallinity in the π-π stacking direction. When the temperature was subsequently decreased, an increase of the conductivity was observed. The partial backward phase transition from the mesophase to the crystalline phase was observed at about 45 °C. The correlation of the conductivity and crystallinity of the thin-film structure confirms that the π-π stacking interaction is the key for enhanced charge transport.

The observation of the monolayer-type phase constitutes an interesting finding for such class of semiconducting molecules. It is noteworthy that previously similar interfacial phase was observed for the case of α,α′-DH5T [31]. Although the microstructure and ability to crystallize are different for DH4T and DH5T, which is probably correlated to the odd-pair effect in the structure of oligothiophenes [35, 38], thin films of both compounds exhibit the presence of the monolayer-type phase in the vicinity of the substrate surface. Therefore, the observed structure provides additional support to the view that such surface-induced polymorphs (cf. e.g., [24, 25]) may constitute a general feature for the whole class of such and similar compounds.

Further work will be clearly needed to correlate the electrical and structural properties of such molecules as a function of deposition conditions and temperature. However, it is already clear that the charge transport can be to a great extent defined by the presence and extent of the described monolayer-type phase.

## Conclusions

A combined in-situ investigation of the structure and electrical properties of thin vacuum-deposited DH4T films was performed to correlate the microstructure, type of the phase, and the charge transport. The initial crystalline structure exhibits a large number of Bragg reflections allowing assigning it to a monoclinic phase. Importantly, the deposited film reveals the high and uniform orientation of the domains. It was found that the D4HT molecules are inclined by 29° with respect to the surface normal. During annealing experiments, a transition from the initial crystalline phase to the mesophase was detected. The structural transformations were found to significantly impact the electrical conductivity measurements at around 85 and 45 °C, which correspond to the transition from the initial crystalline phase to the mesophase and to partial backward transition. In situ correlation of the charge transport and microstructural features confirms that a highly crystalline structure with a strong in-plane π-π orientation is responsible for the highest conductivity. The variable temperature synchrotron studies allowed us to detect a particular nanostructure which can be assigned to a monolayer-type phase presumably stabilized by the substrate surface. The existence of this particular interfacial layer may have important implications for the charge mobility, especially for the case when the measurements are performed in the OFET geometry where one probes the electric properties of a relatively thin layer close to the substrate. Indeed, such monolayer-type phase can be mainly responsible for the conduction properties of the oligothiophene systems at elevated temperatures. Moreover, this finding might constitute a general feature of this class of molecules, which would require revisiting the correlations between the charge mobility and nanostructure.

## Abbreviations

AFM: Atomic force microscopy
D4HT: Dihexyl-quarterthiophene
D5HT: Dihexyl-quinquethiophene
GIXD: Grazing-incidence X-ray diffraction
XRR: X-ray reflectivity
